# A Neuropsychological Test of Belief and Doubt: Damage to Ventromedial Prefrontal Cortex Increases Credulity for Misleading Advertising

**DOI:** 10.3389/fnins.2012.00100

**Published:** 2012-07-09

**Authors:** Erik Asp, Kenneth Manzel, Bryan Koestner, Catherine A. Cole, Natalie L. Denburg, Daniel Tranel

**Affiliations:** ^1^Division of Behavioral Neurology and Cognitive Neuroscience, Department of Neurology, University of Iowa College of MedicineIowa City, IA, USA; ^2^Department of Marketing, University of IowaIowa City, IA, USA; ^3^Department of Psychology, University of IowaIowa City, IA, USA

**Keywords:** prefrontal cortex, deception, advertising, lesion, credulity, false tagging theory, belief, doubt

## Abstract

We have proposed the *False Tagging Theory* (FTT) as a neurobiological model of belief and doubt processes. The theory posits that the prefrontal cortex is critical for normative doubt toward properly comprehended ideas or cognitions. Such doubt is important for advantageous decisions, for example in the financial and consumer purchasing realms. Here, using a neuropsychological approach, we put the FTT to an empirical test, hypothesizing that focal damage to the ventromedial prefrontal cortex (vmPFC) would cause a “doubt deficit” that would result in higher credulity and purchase intention for consumer products featured in misleading advertisements. We presented 8 consumer ads to 18 patients with focal brain damage to the vmPFC, 21 patients with focal brain damage outside the prefrontal cortex, and 10 demographically similar healthy comparison participants. Patients with vmPFC damage were (1) more credulous to misleading ads; and (2) showed the highest intention to purchase the products in the misleading advertisements, relative to patients with brain damage outside the prefrontal cortex and healthy comparison participants. The pattern of findings was obtained even for ads in which the misleading bent was “corrected” by a disclaimer. The evidence is consistent with our proposal that damage to the vmPFC disrupts a “false tagging mechanism” which normally produces doubt and skepticism for cognitive representations. We suggest that the disruption increases credulity for misleading information, even when the misleading information is corrected for by a disclaimer. This mechanism could help explain poor financial decision-making when persons with ventromedial prefrontal dysfunction (e.g., caused by neurological injury or aging) are exposed to persuasive information.

## Introduction

It may seem like a stroke of good luck to be contacted by a Nigerian prince who is in trouble. The individual often claims to have some connection to a large fortune but needs a foreign investor’s help to access it. Victims of this fraud scheme may send thousands of dollars to this individual with the promise of a 10-fold payoff in return. Unfortunately for victims, the reward never arrives.

Fraud, an intentional deception made for personal gain, is a crime and has reached epidemic levels in older adults. An estimated 7.3 million adults 65 years of age or older (20% of older Americans) have been the victims of financial fraud according to a 2010 survey (Infogroup/ORC, 2010). Research has suggested older adults are disproportionally vulnerable to fraud and deception in general (Gaeth and Heath, 1987; Chen and Blanchard-Fields, 2000; Chen, 2002, 2007). However, we remain without an adequate understanding of the elderly individual’s propensity toward credulity when exposed to persuasive messages. Moreover, we still do not understand the neuroanatomical mechanisms which (1) are critical in belief and doubt processes, and (2) might show disproportional dysfunction in connection with age-related increases in credulity. A central goal of our research is to investigate the underlying neuroanatomical mechanisms which are engaged when one becomes dubious or skeptical. The studies highlighted above have indicated that older adults may have impairments in these mechanisms but do not address from a neuroanatomical perspective why older adults are more vulnerable to deception and misleading information, which often results in poor financial decision-making.

Denburg et al. (2007) have indicated that the vulnerability to misleading information in older adults may be linked to an impairment in prefrontal cortex functioning. The structural integrity of the prefrontal cortex is preferentially diminished relative to other brain regions in some older adults (Dempster, 1992; Raz et al., 1997; Pfefferbaum et al., 2005); and there is a decline in frontal lobe functioning beyond the sixth decade of life (West, 1996; Phillips et al., 2002). However, this leaves us with the question of how prefrontal cortex dysfunction results in vulnerability to misleading information. As another way of putting the question, what does the prefrontal cortex do to prevent credulity and gullibility?

To address this question, Asp and Tranel (2012) recently developed the False Tagging Theory (FTT), a neuroanatomically based theoretical model of belief and doubt processes. In brief, the FTT asserts that (1) the process of belief occurs in two stages, mental representation and assessment (Gilbert, 1991); (2) all ideas that are represented are initially believed, but a secondary psychological analysis (assessment) can produce disbelief (or doubt) (Gilbert, 1991; Gilbert et al., 1993); (3) the mental representation of the idea, which is initially believed or regarded as true, must be “tagged” to indicate false value, producing doubt (Gilbert, 1991); (4) the prefrontal cortex is necessary for the “false tag” in the assessment component of belief; and (5) “false tags” are affective in nature, akin to the central tenets of Damasio’s (1994) “somatic marker hypothesis.” Our model suggests that the key function of the prefrontal cortex is “false tagging” which, in the cognitive domain, acts to doubt cognitive representations (which are initially believed). The FTT views the prefrontal cortex as providing a singular function that multiple modalities can access and use (Asp and Tranel, 2012); however, certain prefrontal regions are more inclined to “false tag” for particular modalities, and we suggest that the ventromedial prefrontal cortex (vmPFC) is particularly critical for false tagging cognitive representations. Therefore, the ventromedial portion of the prefrontal cortex is of central interest to the study of cognitive belief and doubt. Other prefrontal regions may also play critical roles in doubting, e.g., acting as a false tagging resource “reserve” (Asp and Tranel, 2012). However, this study will focus exclusively on the vmPFC’s role in the belief and doubt process. The FTT predicts that dysfunction of the vmPFC should result in a “doubt deficit,” consequences of which should be credulity and a tendency to believe inaccurate information. Several preliminary studies have bolstered the theory, including the findings that patients with focal damage to the vmPFC (1) often have a general personality trait that is overconfident, boastful, grandiose, obstinate, and egocentric (Stuss and Benson, 1984; Damasio et al., 2011), indicating a lack of normative doubt; (2) are more gullible to disreputable characters (Damasio, 1994; Croft et al., 2010); and (3) are more likely to believe fundamentalist religious dogma (Asp et al., 2012). Thus, vmPFC patients and older adults may have a vulnerability to believe deceptive or misleading information because vmPFC dysfunction impairs normative doubt.

Under our FTT, beliefs are broad and cover all mental representation, including all cognitive representations such as knowledge, ideas, opinions, attitudes, and rules (Asp and Tranel, 2012). Traditional perspectives of cognitive representation have suggested that these cognitions are like tools in a warehouse; they are actual objects in the brain that can be retrieved and used (Gilbert, 1993). The underlying assumption is that cognitions in these models are static; they are the bits, the 1’s and 0’s, of the mind’s computer. There are several shortcomings to this computational view, most notably, that (1) the mind’s “CPU” (the person getting the tools from the warehouse) must perform homuncular-type operations (e.g., Baddeley, 2002) and (2) cognitions are impotent (Gilbert, 1993). Computational models require a faculty or mechanism (a “CPU”) to do action with static cognitions (the 1’s and 0’s), which cannot produce effects on their own. However, the FTT asserts that all cognitions are beliefs; they are “empowered” intrinsically with simple comprehension (Gilbert, 1993). Thus, when an individual understands a novel proposition, the individual is automatically put in a “state” of belief. Here, the mental representation is not impotent but will induce action, given appropriate circumstances. To avoid every passing idea to be acted on, the vmPFC can doubt or disbelieve cognitions by applying false tags to mental representations. Understanding a cognition is, then, more like the “state” of a shot of an ice hockey player directed at a goalie. If the goalie does not stop the shot (false tag the cognition), the shot will go in the net (a cognition-consistent action will be performed). The belief will be acted upon if not blocked by the vmPFC. In this model, post-rolandic cortices are constantly firing shots and the vmPFC is reliably blocking some percentage of them.

If this logic is applied to a decision-making scenario, each choice that is identified (i.e., understood) is a belief (“if this, then that” cognitions) and “false tags” block disadvantageous or inappropriate choices for the context. False tags (or doubt) via the vmPFC act to select appropriate responses during decision-making by negatively biasing the inappropriate (i.e., “untrue”) representations. Therefore, we propose that dysfunction or damage to the vmPFC has a two-pronged, but intimately related, effect: (1) a tendency toward credulity for deceptive or misleading information; and (2) disadvantageous behavioral decision-making.

The purpose of the present study was to investigate credulity and financial decision-making for misleading information presented in a real-world, ecologically valid paradigm (deceptive advertising) in patients with focal brain damage, with the goal of identifying a systems-level neuroanatomical correlate for these cognitive functions. We chose consumer advertisements which had been deemed deceptive and deliberately misleading by the Federal Trade Commission (FTC), and examined participants’ credulity toward the advertisements. Our theory suggests that when normal individuals are exposed to misleading information they will initially believe the information but then will tend to self-generate doubt from their store of knowledge and experience. To examine the interaction between “self-generated” doubt and doubt from explicit information, we created two types of misleading ads: (1) “deceptive-uncorrected” ads, which are misleading and do not have any explicit information that may induce doubting, and (2) “deceptive-corrected” ads, which are misleading but do have an end disclaimer which should induce doubting. The FTT asserts that doubt is mediated by the vmPFC. Thus, we hypothesized that patients with damage to the vmPFC, compared to patients with brain damage outside the prefrontal cortex and healthy individuals, (1) would be more likely to believe the misleading aspects in both the “deceptive-uncorrected” and the “deceptive-corrected” ads, and (2) would indicate higher intention to purchase the products featured in both types of ads.

## Materials and Methods

### Participants

**Figure 1:**
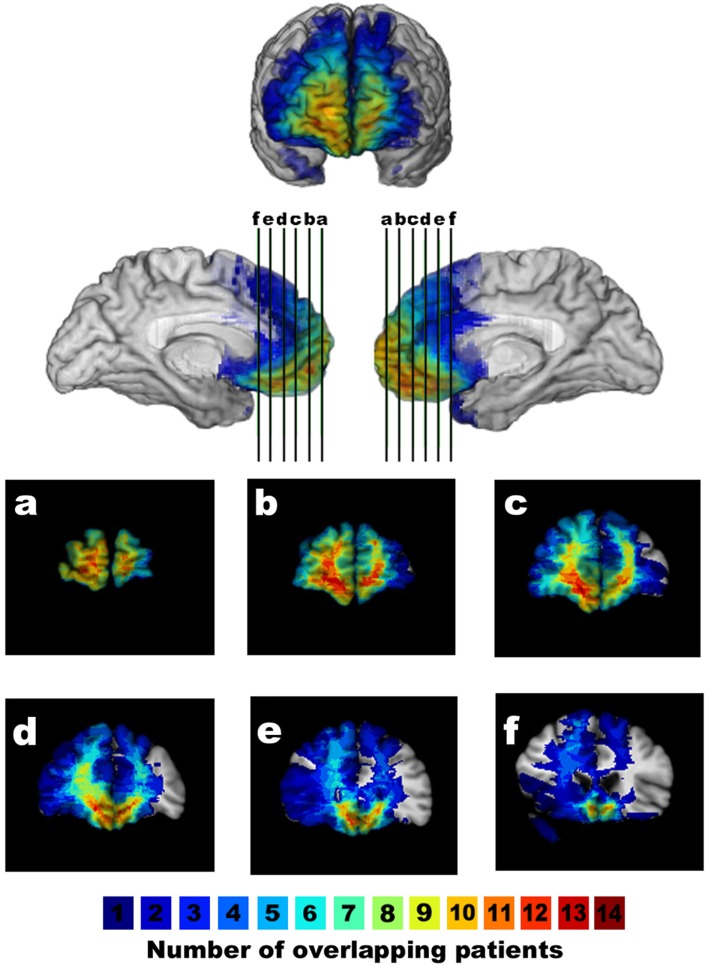
**Lesion overlap of patients with ventromedial prefrontal cortex lesions**. The overlap map shows the lesions of vmPFC patients displayed in anterior/mesial views and coronal slices (a–f, with the right hemisphere on the left in the coronal views). The color bar indicates the number of overlapping lesions per voxel.

**Figure 2:**
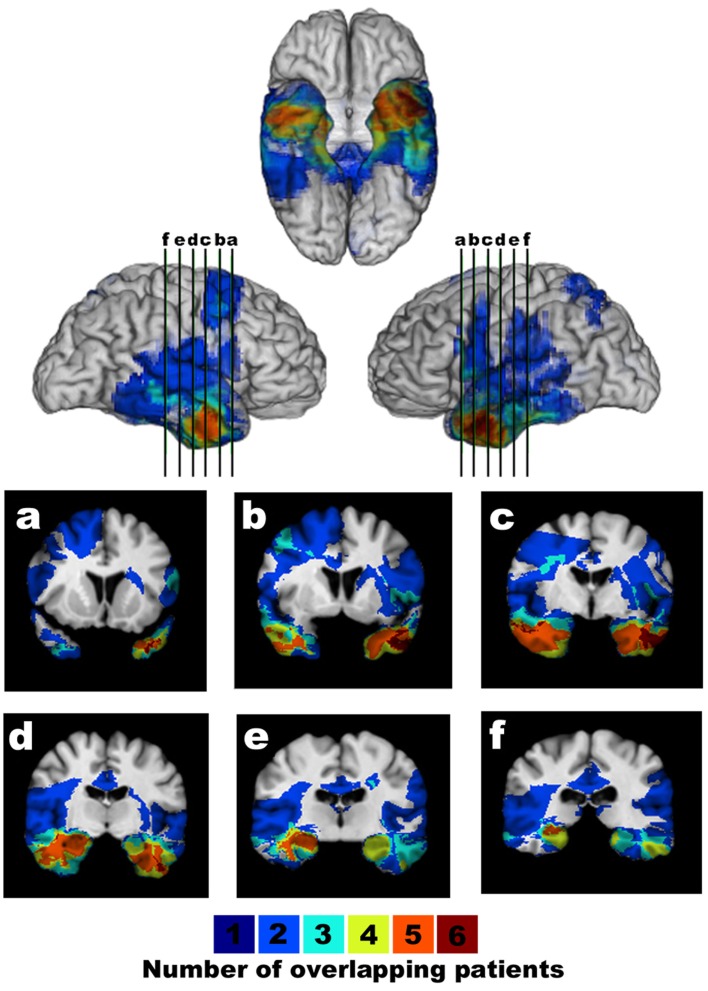
**Lesion overlap of brain damage comparison patients**. The overlap map shows the lesions of BDC patients displayed in ventral/lateral views and coronal slices (a–f, with the right hemisphere on the left in the coronal views). The color bar indicates the number of overlapping lesions per voxel.

We studied 39 individuals with adult-onset brain lesions from the Patient Registry of the Division of Behavioral Neurology and Cognitive Neuroscience at the University of Iowa. The etiologies of the lesions included cerebrovascular disease (*n* = 21), surgical resection for treatment of a meningioma or seizure control (*n* = 15), and focal contusions from trauma (*n* = 3). In connection with their enrollment in the Patient Registry, the brain damaged patients have been extensively characterized neuropsychologically and neuroanatomically, using standard protocols of the Benton Neuropsychology Laboratory and the Laboratory of Brain Imaging and Cognitive Neuroscience (Tranel, 2007). Eighteen patients had damage to the vmPFC and were classified into our vmPFC group (Figure 1); while 21 patients had lesions outside the prefrontal cortex and were classified into our brain damaged comparison group (BDC; Figure 2). Patients with prefrontal cortex damage to areas primarily outside the ventromedial regions were excluded from analysis. While other prefrontal areas are predicted to have a role in “false tagging” it is beyond the scope of this study to address more specific relationships within the prefrontal cortices. All neuropsychological and neuroanatomical data were collected in the chronic phase of recovery, at least 3 months post-lesion onset. We also included a normal comparison group (*n* = 10) which was comprised by individuals of similar age and education to our patient groups. BDC patients were slightly younger and had more females relative to males than our vmPFC group and normal group (Table 1), so we corrected for these differences in the main analyses. VMPFC patients had significantly larger lesions relative to BDC patients (Table 1); thus, a secondary analysis directly comparing credulity in the BDC and vmPFC groups was conducted to account for lesion size. Participants with significant language, memory, or visuoperceptual deficits which might impair their ability to adequately complete the task were excluded. We excluded patients with significant aphasia (defined as two standard deviations below the mean on the Boston Naming Test or the Token Test), reading deficits (defined as two standard deviations below the mean on the Iowa Chapman Reading Test), memory deficits (defined as two standard deviations below the mean on the Auditory Verbal Learning Test delayed recall or the Complex Figure Test delayed recall), or visuoperceptual impairments (defined as two standard deviations below the mean on the Facial Recognition Test). There were no significant differences between vmPFC and BDC patients on the various neuropsychological measures (Table 2). All participants were free from mental retardation, learning disabilities, psychiatric disease, substance abuse, and dementia. Participants gave informed consent approved by the Institutional Review Board of the University of Iowa.

**Table 1:** **Demographic and neuroanatomical data**.

	vmPFC	BDC	Normal
Number	18	21	10
Age (SD)*	60.4 (10.6)	50.2 (11.0)	60.7 (8.9)
Education (SD)	13.8 (2.7)	14.3 (2.3)	15.8 (3.0)
Sex**	12 M; 6 F	6 M; 15 F	7 M; 3 F
Lesion size (SD)^†^	51.7(40.3)	21.9 (15.3)	NA

*Age and education are presented in years; lesion size is presented in cubic centimeters*.

*BDC patients were significantly younger than vmPFC patients and normal participants.

**The BDC group had a significantly lower proportion of males relative to females than the vmPFC and normal groups.

*^†^VMPFC lesions were significantly larger than BDC lesions*.

**Table 2:** **Neuropsychological data for the lesion groups**.

	vmPFC	BDC
WAIS III – FSIQ (SD)	108.5 (16.8)	104.7 (11.5)
WRAT – Read (SD)	99.4 (9.8)	96.6 (8.2)
AVLT – 30 min recall (SD)	8.5 (3.6)	9.2 (2.9)
CFT – 30 min recall (SD)	20.0 (7.5)	17.2 (5.4)
TMT – Part B (SD)	76.7 (34.9)	77.8 (43.2)
WCST – Pers. Errors (SD)	22.1 (24.7)	12.6 (8.1)

*WAIS-III, Wechsler Adult Intelligence Scale-III scores (FSIQ, full-scale IQ). WRAT, Wide Range Achievement Test scores (Read, Reading Standard Score). AVLT, Auditory Verbal Learning Test scores (an index of memory function at 30 min). CFT, Complex Figure Test recall scores (an index of memory function at 30 min). TMT, Trail Making Test Part B scores, an index of divided attention and multi-tasking. WCST, Wisconsin Card Sorting Test Perseverative Errors, an index of reasoning and concept formation (executive functioning). There were no significant differences between the groups for any of the neuropsychological tests*.

### Stimuli and procedure

Participants were given a booklet that consisted of eight advertisements that one might encounter in a magazine or newspaper. Each ad was based on real-world misleading advertisements as deemed by the rulings of the FTC, as shown in *FTC Decisions* (Federal Trade Commission, 1991) and *Complying with the Made in USA Standard* (Federal Trade Commission Bureau of Consumer Protection, 1998). These advertisements were misleading for a number of reasons, ranging from the withholding of crucial information about the product to the use of biased graphs. For example, in an advertisement for “Legacy Luggage,” the original misleading version had the headline “Legacy brings you the finest American Quality luggage.” The FTC stated that any advertisement that has “American Quality” on it conveys that the item in question was made in the U.S.A. In fact, the luggage was actually not made in the U.S.A., but instead was manufactured in Mexico, and then inspected in Tennessee, and thus was misleading. Three ads were classified as “deception-uncorrected” and were left unchanged from the FTC-ruled “misleading advertisement” classification. However, “deception-uncorrected” ads assume that all individuals have a similar knowledge base regarding potential objections to the misleading portions of the ads. This assumption leaves open the possibility that some individuals may not have, or cannot access, cognitions that should induce doubting. To address this issue, we developed “deception-corrected” ads which provide explicit information that should induce doubting, by modifying three misleading ads with a disclaimer at the end of the ad. The disclaimers in the “deception-corrected” ads specifically rebutted the misleading aspect of the ad. For example, in an advertisement for “NatureCure,” the misleading ad describes a natural pain reliever that provides relief from headaches “without the side effects of over-the-counter pain relievers.” The end disclaimer refutes this claim by noting, “This product can cause nausea in some consumers when taken regularly.” Thus, for the “deception-corrected” ads, all participants were given the same specific knowledge to doubt the misleadingly advertised claim. Finally, there were two “filler” advertisements, one placed at the beginning and the other at the end of the booklet. These were used to help buffer against primacy and recency effects, and were not scored. This left six critical advertisements: three “deception-uncorrected” and three “deception-corrected.” Each ad highlighted a distinct product: the “deception-uncorrected” stimuli advertised a doll, luggage, and a vitamin supplement drink; the “deception-corrected” stimuli advertised a car, a pain reliever, and mutual funds. Participants read over the advertisements at their own pace and when finished, they were given a paper questionnaire which assessed participant reactions to each advertisement and product. Participants could not refer back to the advertisements during the questionnaire; instead, they needed to recall their impressions of each product from memory. Readers who are interested in knowing more about the advertising stimuli and their development may contact the senior author via email.

Two critical dimensions were assessed for each advertisement: (1) credulity toward the misleading aspect of the advertisement, and (2) purchase intention, i.e., how likely was the participant to buy each item should it become available in their area. The credulity measure asked “What do you believe to be true about this product?” and was assessed on a Likert scale, anchored at each end by a belief about the product being advertised. The Likert scales on the questionnaire contained no numerals but had 7 empty spaces (of equal size) between the two anchors. Participants marked the empty space they considered appropriate. Numbers for the Likert scale were added *post hoc*, and ranged from 1 to 7, with lower values reflecting increased belief in the misleading aspects of the ads and higher values reflecting increased skepticism for the misleading aspects of the ads. For example, the previously mentioned Legacy Luggage advertisement dealt with whether or not the luggage was made in the U.S.A. The credulity question concerning that advertisement was anchored at space 1 by “The Legacy Luggage Set is made in the United States” and at space 7 by “The Legacy Luggage Set is NOT made in the United States.” The purchase intention measure was assessed by asking, “What is the probability that you would buy the product when it becomes available in the area?” Participants’ responses were measured on a Likert scale, anchored by “Likely” and “Unlikely.” This scale ranged from 1 to 5, with 1 reflecting a higher intention to purchase the item and 5 reflecting a lower intention to purchase the item.

### Neuroanatomical analysis

The neuroanatomical analysis of the vmPFC and BDC patients (Figures 1 and 2) was based on magnetic resonance data for 30 patients and on computerized tomography data for 9 patients. Using Brainvox (Frank et al., 1997), each patient’s lesion was reconstructed in three dimensions for the different groups. The lesion contour for each patient was manually warped into a normal template brain using the MAP-3 method. The overlap of lesions in these volumes, calculated by the sum of *n* lesions overlapping at any single voxel, is color-coded in Figures 1 and 2. As Figure 1 shows, the greatest overlap of vmPFC patient lesions is in the mesial orbital region, especially the anterior half of the gyrus rectus. The greatest overlap of lesions in Figure 2 is in the inferior temporal lobes. No BDC lesions encompassed the prefrontal cortex.

## Results

All statistical *t*-tests are one-tailed in accordance with our directional predictions.

### Credulity dimension

**Figure 3:**
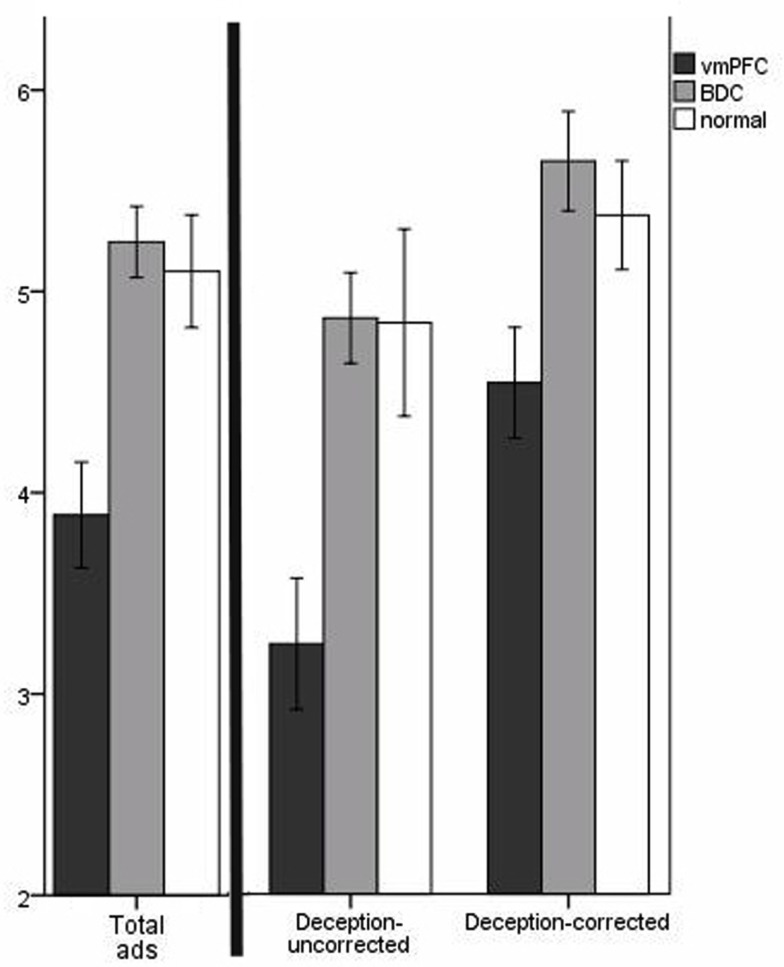
**Mean belief scores for misleading ads**. The scale is from 1 to 7 (*y*-axis), with lower values corresponding to increased belief in misleading aspects of the ads and higher values corresponding to increased skepticism for misleading aspects of the ads. Error bars indicate SEM. The graph on the left of the black bar represents results for all six misleading ads; the graph on the right of the black bar breaks the results down according to “deception-uncorrected” and “deception-corrected” ads. For all the ads, vmPFC patients had more credulity than BDC patients and normal comparison participants.

The first question we addressed was whether patients with vmPFC damage were more likely to be credulous to the misleading advertising overall (including both “deception-uncorrected” and “deception-corrected”). VMPFC patients were more credulous to the misleading advertising (*M* = 3.89, SD = 1.11) than BDC patients (*M* = 5.25, SD = 0.81) and normal participants (*M* = 5.10, SD* *= 0.88; Figure 3). Because BDC patients were slightly younger and had more females relative to males (Table 1), we ran an ANCOVA with age and sex as covariates. The covariate, age, was not significantly related to credulity, *F*(1,44) = 0.01, *p* = 0.93; and the covariate, sex, was also not significantly related to credulity, *F*(1,44) = 0.01, *p* = 0.92. There remained a significant difference in group credulity after controlling for the covariates, *F*(2,44) = 9.26, *p *< 0.001. Planned contrasts revealed that vmPFC patients were more credulous than BDC patients, *t*(44) = 3.17, *p *= 0.002, and normal participants, *t*(44) = 3.88, *p *< 0.001.

Ventromedial prefrontal cortex patients had significantly larger lesions that BDC patients (Table 1) and there was a modest correlation between lesion size and credulity to the ads, *r* = −0.31, *p* = 0.06. Thus, a secondary analysis was conducted to directly examine the influence of lesion size on the credulity measure in the two patient groups. The covariate, lesion size, was not significantly related to credulity, *F*(1,36) = 0.12, *p* = 0.74. There remained a significant difference in group credulity after controlling for the covariate, *F*(1,36) = 13.70, *p* = 0.001.

Splitting the overall credulity results into the three “deception-uncorrected” ads and the three “deception-corrected” ads helps clarify our initial analysis. VMPFC patients were more credulous to the “deception-uncorrected” ads (*M* = 3.24, SD* *= 1.38) than BDC patients (*M* = 4.86, SD = 1.03) and normal participants (*M* = 4.83, SD* *= 1.47; Figure 3). There was a significant difference for group credulity on the “deception-uncorrected” ads, *F*(2,46) = 9.25, *p* < 0.001. Planned contrasts revealed that vmPFC patients were more credulous than BDC patients, *t*(46) = 4.18, *p *< 0.001, and normal participants, *t*(46) = 3.20, *p *= 0.002. vmPFC patients were also more credulous to the “deception-corrected” ads (*M* = 4.54, SD = 1.17) than BDC patients (*M* = 5.64, SD* *= 1.13) and normal participants (*M *= 5.37, SD = 0.85; Figure 2). There was a significant difference for group credulity on the “deception-corrected” ads, *F*(2,46) = 5.06, *p *= 0.01. Planned contrasts revealed that vmPFC patients were more credulous than BDC patients, *t*(46) = 2.90, *p* = 0.003, and normal participants, *t*(46) = 1.92, *p *= 0.03. These data suggest that higher credulity toward misleading ads in vmPFC patients was obtained even when explicit disclaimers should induce doubt for the misleading information.

A secondary repeated measures ANOVA analysis was conducted to see if the “correction” in the ads had a significant main effect. While the participants were generally more skeptical for the “deception-corrected” ads, there was not a significant main effect of “correction,” *F*(1,44) = 0.15, *p* = 0.70. In addition, the groups were not significantly different in the way they were affected by the presence of corrective information, *F*(2,44) = 1.02, *p* = 0.37. Thus, the corrective information did help the vmPFC patients increase their skepticism similarly to the comparison groups.

### Purchase intention dimension

**Figure 4:**
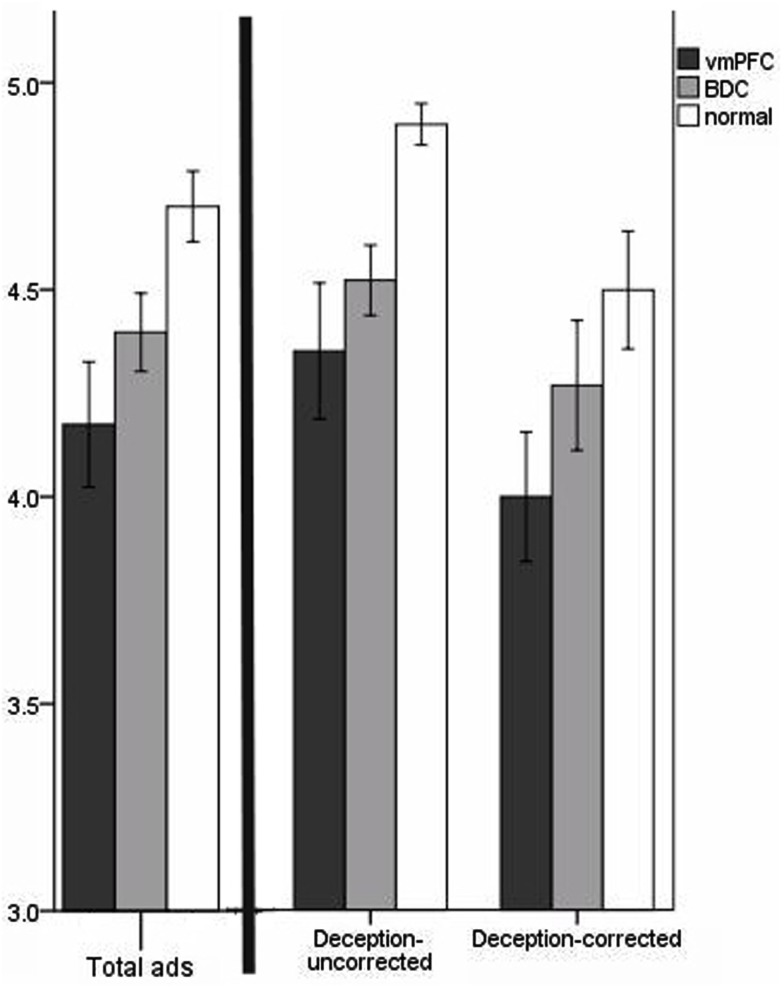
**Mean purchase intention scores for misleading ads**. The scale is from 1 to 5 (*y*-axis). Lower values reflect increased purchase intention for the products misleadingly advertised, and higher values reflect decreased purchase intention. Error bars indicate SEM. The graph on the left of the black bar represents results for all six misleading ads; the graph on the right of the black bar breaks the results down according to “deception-uncorrected” and “deception-corrected” ads. For all the ads, vmPFC patients had higher purchase intention than BDC patients and normal comparison participants for the products in the misleading ads.

For the purchase intention dimension, we addressed first whether vmPFC patients overall were more likely to have intent to purchase the advertised products. VMPFC patients had higher purchase intention for the misleadingly advertised products (*M* = 4.17, SD = 0.64) than BDC patients (*M *= 4.40, SD = 0.43) and normal participants (*M *= 4.70, SD = 0.27; Figure 4). Again, we used age and sex as covariates in an ANCOVA analysis. The covariate, age, was not significantly related to purchase intention, *F*(1,44) = 0.93, *p* = 0.34; and the covariate, sex, was also not significantly related to purchase intention, *F*(1,44) = 2.17, *p *= 0.15. There remained a significant difference in purchase intention after controlling for the covariates, *F*(2,44) = 3.75, *p* = 0.03. Planned contrasts revealed that vmPFC patients had significantly higher purchase intention than normal participants, *t*(44) = 2.74, *p *= 0.005; vmPFC patients did not have significantly higher purchase intention than the BDC patients, *t*(44) = 1.13, *p* = 0.13. Lesion size was uncorrelated with purchase intention, *r* = 0.04, *p* = 0.81.

When the purchase intention data were divided into “deception-uncorrected” and “deception-corrected,” the results indicated that vmPFC patients had higher purchase intent for the “deception-uncorrected” ads (*M* = 4.35, SD = 0.70) than BDC patients (*M* = 4.52, SD* *= 0.39) and normal participants (*M* = 4.90, SD = 0.16; Figure 4). There was a significant difference for group purchase intention on the “deception-uncorrected” ads, *F*(2,46) = 3.86, *p *= 0.03. Planned contrasts showed that vmPFC patients had higher purchase intention than normal participants, *t*(20) = −3.18, *p *= 0.003; but did not significantly differ from BDC patients, *t*(26) = −0.93, *p* = 0.18. VMPFC patients also had higher purchase intent to the “deception-corrected” ads (*M* = 4.00, SD = 0.67) than BDC patients (*M* = 4.27, SD = 0.72) and normal participants (*M* = 4.50, SD = 0.45; Figure 3). However, group differences on “deception-corrected” ads for purchase intention did not reach significance, *F*(2,46) = 1.99, *p *= 0.14. Planned contrasts revealed that vmPFC patients had significantly higher purchase intention than normal participants, *t*(46) = −1.94, *p* = 0.03; but did not significantly differ from BDC patients, *t*(46) = −1.28, *p* = 0.11.

## Discussion

Our findings support the hypothesis that credulity toward misleading information can result from damage to the vmPFC. Patients with vmPFC damage tended to (1) believe misleading advertisements, and (2) show higher intent to purchase the products featured in the misleading advertisements, relative to patients with brain damage outside of the prefrontal cortex and normal comparison participants. Remarkably, the pattern of credulity results was evident even when vmPFC patients were given specific information that rebuts the misleading claim. This suggests that the deficiency in vmPFC patients is specific to the doubt process, not a lack of knowledge regarding misleading information. Thus, the results indicate that given a deceptive ad (with or without a disclaimer) vmPFC patients are more credulous. The disclaimer did increase skepticism in vmPFC patients (similarly to the comparison groups) but overall the disclaimer did not produce normative skeptical levels in vmPFC patients. Thus, there is a deficiency in skepticism generally, even when specific rebutting knowledge cues a doubting process.

The conclusion that damage to the vmPFC causes an increase in credulity to misleading information is bolstered by the facts that (1) brain damage, *per se*, when outside of the prefrontal cortex, does not account for the results (as evident from the BDC data); (2) demographic variables such as age, education, or sex, *per se*, do not account for the results; and (3) general cognitive functioning, such as intelligence, memory, reading performance, or executive functioning, *per se*, does not account for the results. Instead, the vmPFC patients’ deficit in skepticism to the misleading information is specific to their lesion location and is not accounted for by generally poor cognitive functioning.

The vmPFC patients did have larger lesions relative to the BDC patients (Table 1). However, it is unlikely that lesion size, *per se*, influenced the credulity or purchase intention results. A detailed analysis revealed: (1) lesion size as a covariate was not significantly related to credulity, and (2) when the patients were ranked on the credulity measure (the total ads together), the top 5 most credulous vmPFC patients (*M* = 28.8, SD = 15.5) actually had a slightly smaller mean lesion size than the bottom 5 least credulous BDC patients (*M* = 29.6, SD* *= 23.7). Moreover, an appropriate lesion size measure interpretation must be understood in the context of the region which is damaged. Small lesions to critical structures such as the amygdala, hippocampus, or thalamic nuclei may critically impair a variety of functions, while a similar size lesion to the relatively large and uniform vmPFC may not have similar functional disruptions. Thus, lesion size, *per se*, while different between the two groups, was unlikely to contribute to the increased credulity and purchase intent in the vmPFC group relative to the BDC group, i.e., the deficit is specific not to lesion size but to lesion location.

Our findings support the FTT, which posits that the prefrontal cortex is critical in mediating doubt (Asp and Tranel, 2012), and thus damage to the critical ventromedial region of the prefrontal cortex should result in a “doubt deficit.” While it has been noted that ventromedial prefrontal patients are often vulnerable to shady business ventures and snake-oil salesmen (Damasio, 1994), the current study provides the first direct evidence beyond anecdotal reports that damage to vmPFC increases credulity. Indeed, this specific deficit may explain why highly intelligent vmPFC patients can fall victim to seemingly obvious fraud schemes. Warnings from friends and family often go unheeded and vmPFC patients’ susceptibility can result in bankruptcy if they continue to make their own financial decisions. Moreover, in the acute phase of recovery following vmPFC damage, patients often confabulate and are markedly suggestible (Berlyne, 1972) to other individuals and, on rare occasions, even to the environment around them (Lhermitte, 1986). Taken together, this evidence indicates that the vmPFC is a critical neural structure preventing unwarranted belief toward unscrupulous companies or individuals who try to bilk one’s money.

In our study, we gave novel external persuasive information to vmPFC patients and found that they tend to be credulous to that information. However, as suggested in the Introduction, vmPFC patients can also be obstinate and bull-headed toward novel information. Intuitively, it may appear contradictory that an individual can both be credulous and rigidly obstinate to information. Yet, this is the strange state often exhibited by patients with vmPFC damage. We hypothesize that the critical factor determining the easy acceptance or rigid rejection of information in vmPFC patients is whether the cognitive representation is initially generated by external or internal information. If the cognitive representation is initially generated by external information (as in the present study), it is believed, but then it fails to be falsified by comparisons with extant mental information. Thus, the new information is not doubted, and credulity ensues. If the cognitive representation is initially generated by internal information, it is believed, but then it fails to be falsified by comparisons with new external information. Thus, the old information is not doubted, and a pertinacious belief is evinced. This suggests the initial cognition is always first believed and it is the comparison and falsification to other beliefs that is disrupted. vmPFC patients, then, should have “compartmentalized minds,” where discordant ideas are rarely compared and falsified with one another. Indeed, vmPFC patients tend to be high in authoritarianism (Asp et al., 2012), a trait highlighted by a capacity to hold mutual agreement of contradictory ideas (Altemeyer, 1996). vmPFC patients are also prone to pathological confabulation, where they truly believe their (sometimes florid) assertions, even though contradictory evidence to these assertions is salient and obvious (Gilboa and Moscovitch, 2002).

Our results also indicated that vmPFC patients had higher intention to purchase the misleadingly advertised products than BDC or normal comparisons. Undoubtedly, other, independent factors outside the study’s design likely have stronger influences during an actual purchase decision process (e.g., the usefulness of the product and available financial means for an individual), than a single misleadingly advertised product aspect. These independent factors may have differentially affected the purchase intention data; e.g., the participants gave higher purchase intention ratings to the “deception-corrected” ads compared to the “deception-uncorrected” ads. Thus, because the experimental design used different (and unmatched) products across the types of ads, other issues such as usefulness and monetary concerns probably factored greater in the participants’ purchase intention.

However, it is notable that vmPFC patients had the highest purchase intent of any group. VMPFC patients are notorious for their poor decision-making in financial and social situations. They often claim that an inappropriate decision “feels right”; and there is substantial evidence suggesting that vmPFC patients lack affective signals which normally steer individuals toward advantageous decisions (Damasio, 1994; Bechara et al., 1996, 2000). The FTT asserts that the cognitive process which selects the item eventually chosen from a decision-making process is governed by doubt (or false tags) which are affective in nature (Asp and Tranel, 2012). As an individual mentally represents each potential choice, the vmPFC acts to “doubt” or to negatively bias the inappropriate or undesirable representations away from a behavioral action. Appropriate or desirable response selection, then, is the result of the “fittest” choice representation; i.e., the representation with the least negative biases (or false tags) attached to it. Here, we suggest the cognition to purchase a specific item is a belief and individuals must “false tag” this belief with other extant cognitive information. For instance, in regard to the “Legacy Luggage” we propose that normal individuals believe the initial cognition “I will purchase the Legacy Luggage” but then “falsify” that cognition with discordant extant cognitions, e.g., “I just bought new luggage” or “I don’t have time to go on any trips.” Other product aspects (including the misleading aspects) may play a role in the purchase decision, e.g., “It is made in the US” (strengthening the belief) or “The color of the luggage is unappealing” (increasing false tags) as well. Although we cannot specify what potential cognitions individuals may offer for falsification, in this decision-making scenario, vmPFC patients should be more likely to intend to purchase advertised products. Our results provide some initial evidence for this view.

We believe our results have implications that extend beyond unethical marketing campaigns, although they do directly impact marketing ethics in brain damaged individuals. This study adds to the growing evidence that belief and disbelief are not governed by balanced cognitive processes (Gilbert, 1991). Belief is first, easy, inexorable with comprehension of any cognition, and substantiated by representations in the post-rolandic cortex. Disbelief is retroactive, difficult, vulnerable to disruption, and mediated by the vmPFC. This asymmetry in the process of belief and doubt suggests that false doctrines in the “marketplace of ideas” (Mill, 1975) may not be as benign as is often assumed (Gilbert et al., 1993). Indeed, normal individuals are prone to misleading information, propaganda, fraud, and deception (Zuckerman et al., 1981; Gilbert, 1991), especially in situations where their cognitive resources are depleted. In our theory, the more effortful process of disbelief (to items initially believed) is mediated by the vmPFC; which, in old age, tends to disproportionally lose structural integrity and associated functionality. Thus, we suggest that vulnerability to misleading information, outright deception, and fraud in older persons is the specific result of a deficit in the doubt process which is mediated by the vmPFC.

To conclude, the present findings suggest that the vmPFC is a critical neural substrate for psychological doubt affecting post-rolandic representations. Damage to the vmPFC disrupts a “false tagging mechanism” which normally produces doubt and skepticism for cognitive representations.

## Conflict of Interest Statement

The authors declare that the research was conducted in the absence of any commercial or financial relationships that could be construed as a potential conflict of interest.
